# Supplementary material for: Hippocampal cells segregate positive and negative engrams
Source: Commun Biol. 2022 Sep 26;5:1009. doi: 10.1038/s42003-022-03906-8 (PMC9512908; doi:10.1038/s42003-022-03906-8)
Supplement: Supplementary file 1 — Supplementary Information [file 42003_2022_3906_MOESM1_ESM.pdf]

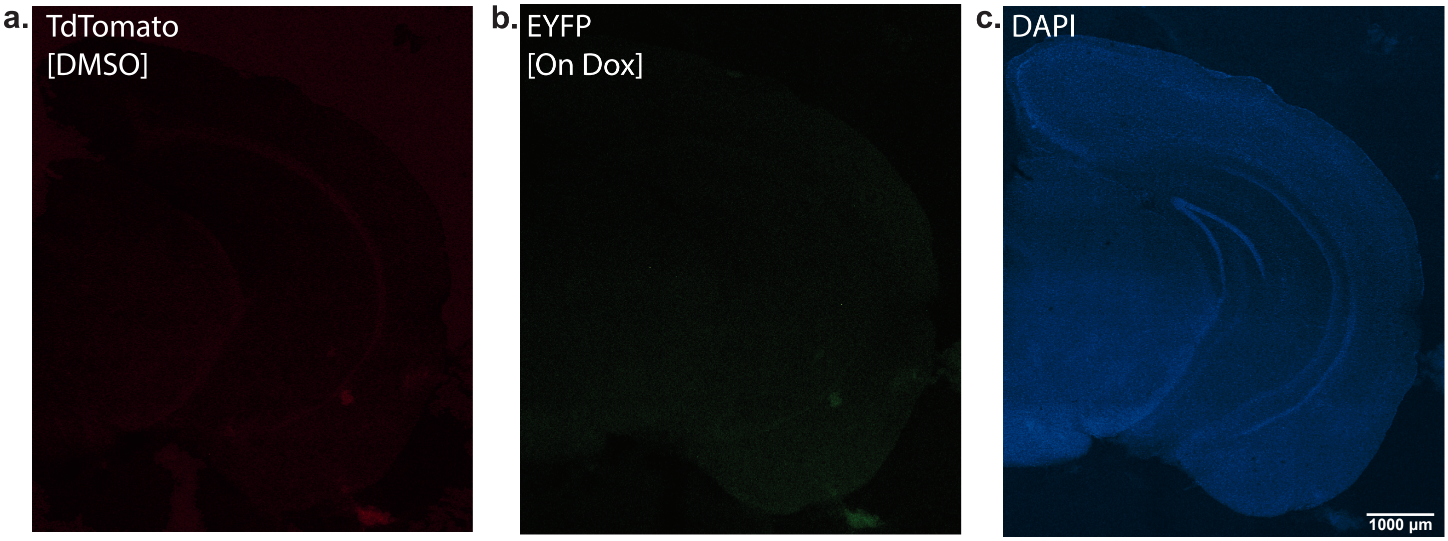
a.
TdTomato
[DMSO]
b.
EYFP
[On Dox]
c.
DAPI
1000 µm

Supplementary Figure 1: Representative image of TRAP2 ‘leakiness’ in vHPC. a, Representative image of TdTomato expression in mouse with DMSO injection instead of 4-OHT. **b,** EYFP expression in the same mouse while remaining on DOX continuously. **c,** DAPI.

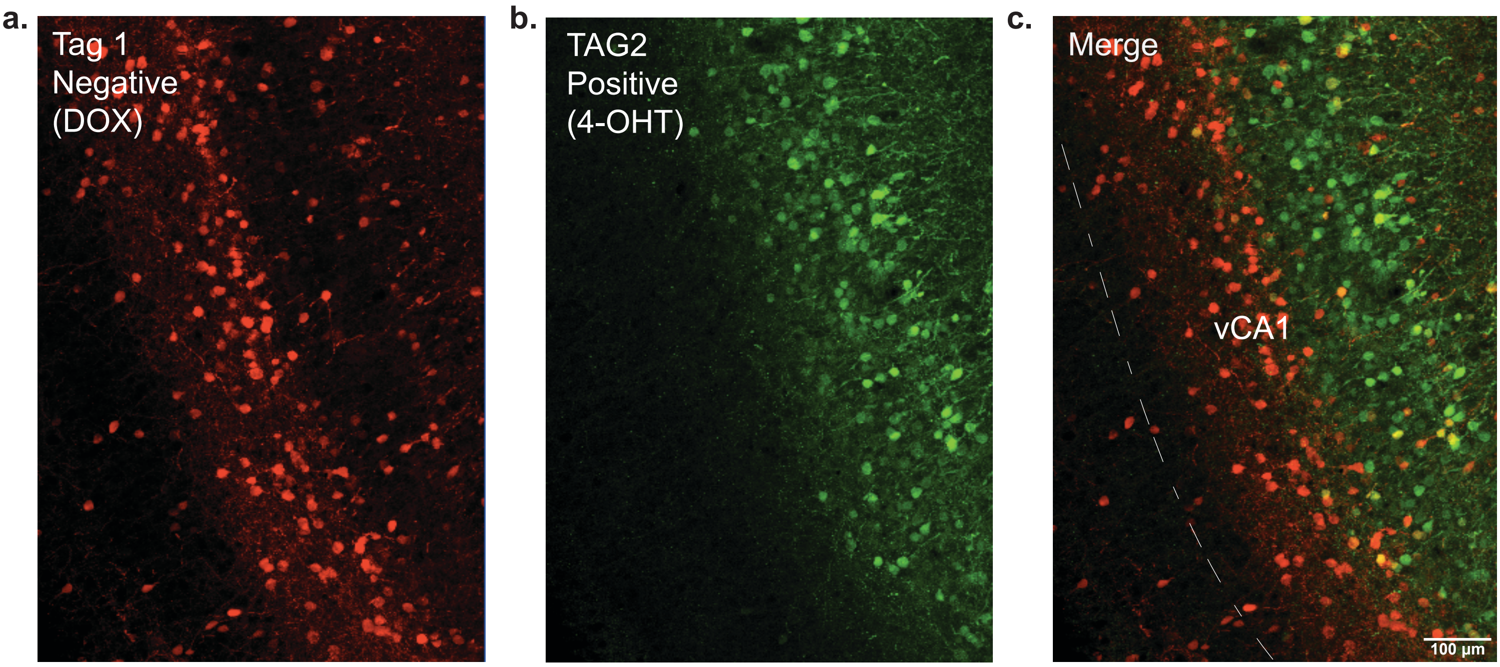
a.
Tag 1
Negative
(DOX)
b.
TAG2
Positive
(4-OHT)
c.
Merge
vCA1
100 µm

Supplementary Figure 2: Anterior-posterior vCA1 segregation of appetitive and aversive engrams occurs regardless of experience timing. Here, the mouse was first taken off dox and experienced four foot shocks at 2 seconds each, 4 times. 48 hours later, the mouse was allowed to interact with a female mouse to tag the appetitive experience with 4-OHT. As shown in figures **a, b,** and **c,** we still see a clear segregation of appetitive and aversive engrams across vCA1, similar to the segregation shown in Fig 1c-d.

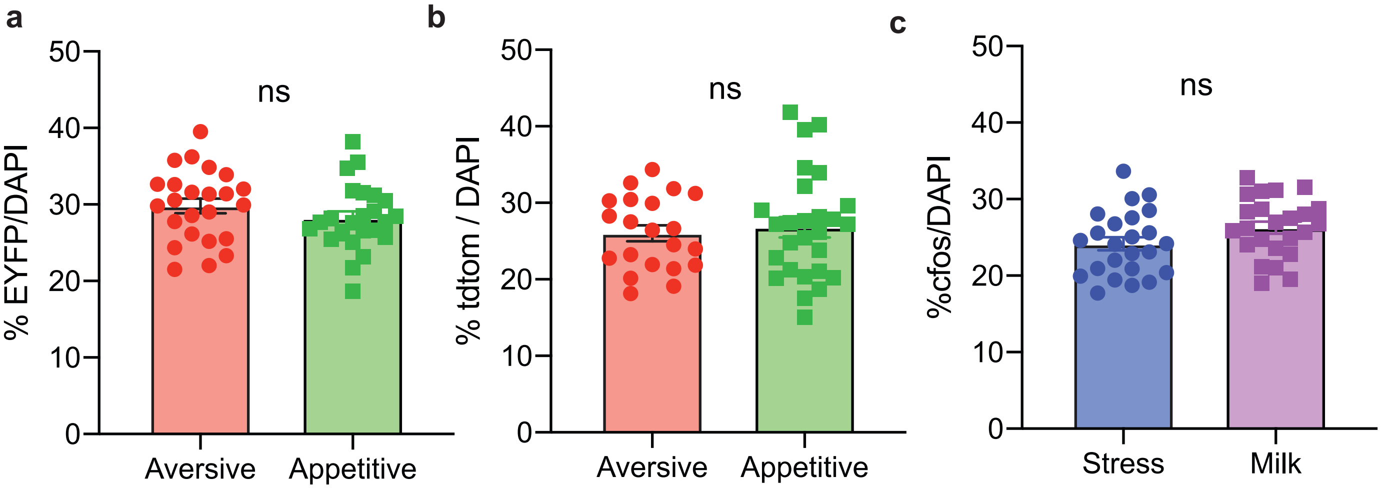
a
b
c
ns
ns
ns
% EYFP/DAPI
% tdtom / DAPI
%cfos/DAPI
0
10
20
30
40
50
Aversive
Appetitive
Aversive
Appetitive
Stress
Milk

Supplementary Figure 3: Cell recruitment based on valence type and cfos overlap measures. a, No significant (ns) difference in % overlap of EYFP over DAPI for shock vs female tags ($t=1.242$, $df=46$; $p=0.2246$). **b,** % No significant (ns) difference in % overlap of TdTomato over DAPI for shock vs female tags ($t=0.4359$, $df=46$; $p=0.665$). **c** No significant (ns) difference in % overlap of cfos over DAPI for shock vs sweetened condensed milk tags ($t=1.840$, $df=46$; $p=0.0722$, mean $\pm$ SEM in bar graphs unless otherwise noted)

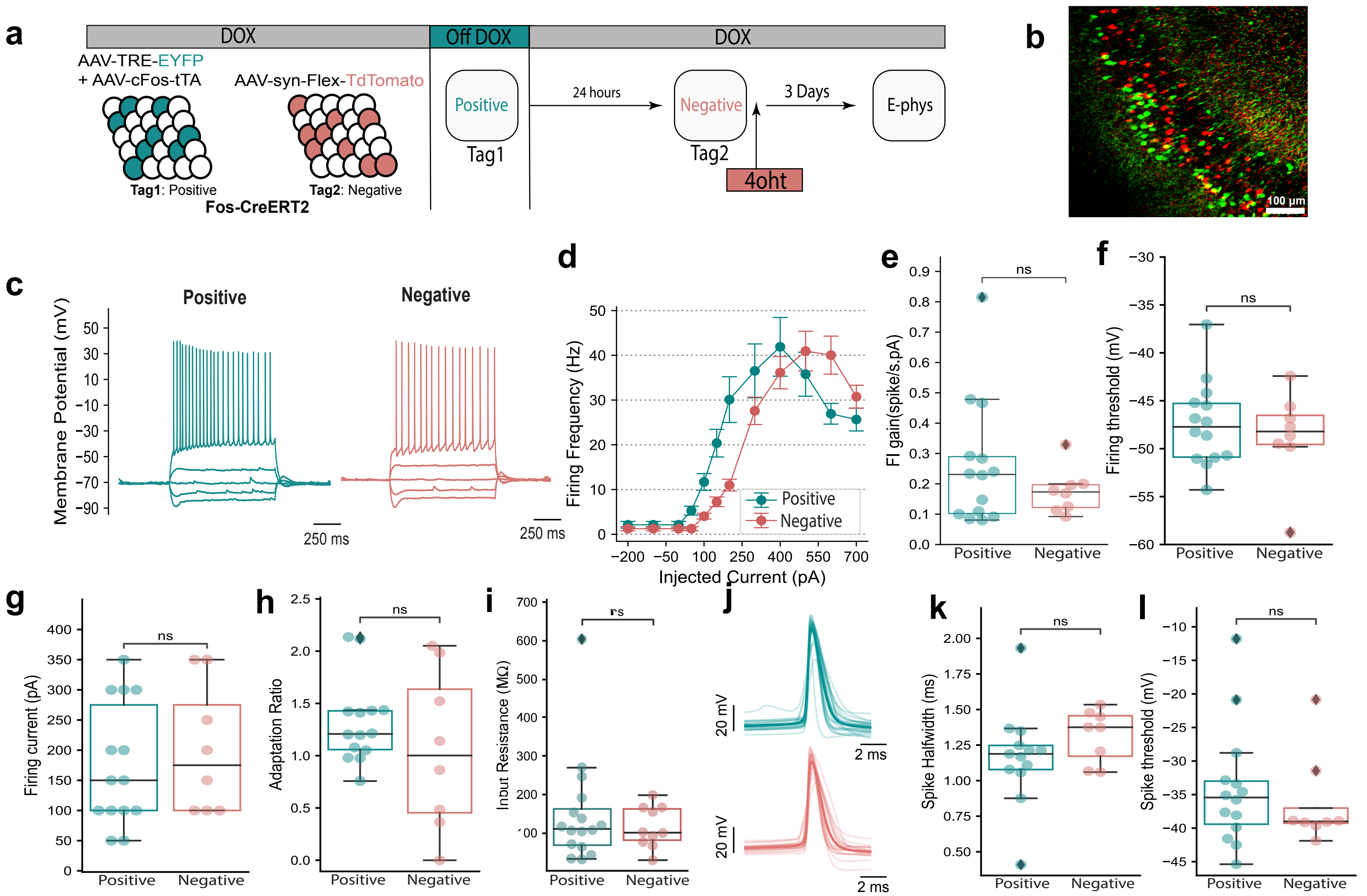
a
DOX
Off DOX
DOX
AAV-TRE-EYFP
+ AAV-cFos-tTA
AAV-syn-Flex-TdTomato
Tag1: Positive
Tag2: Negative
Fos-CreERT2
Positive
Tag1
24 hours
Negative
Tag2
4oht
3 Days
E-phys
b
100 µm
c
Positive
Negative
Membrane Potential (mV)
250 ms
250 ms
d
Firing Frequency (Hz)
Injected Current (pA)
Positive
Negative
e
FI gain(spike/s.pA)
ns
Positive
Negative
f
Firing threshold (mV)
ns
Positive
Negative
g
Firing current (pA)
ns
Positive
Negative
h
Adaptation Ratio
ns
Positive
Negative
i
Input Resistance (MΩ)
ns
Positive
Negative
j
20 mV
2 ms
20 mV
2 ms
k
Spike Halfwidth (ms)
ns
Positive
Negative
l
Spike threshold (mV)
ns
Positive
Negative

Supplementary Figure 4: **Negative and positive valenced cells in the ventral hippocampus share similar electrophysiological characteristics. a,** Fos-CreERT2 were injected with a virus cocktail of AAV-Flex-DIO-TdTomato, AAV9-c-Fos-tTA and AAV9-TRE-EYFP to tag either negative or positive experiences within the same animal for slice electrophysiology. **b,** Representative image of cells processing a negative (red) or positive (green) experience. **c,** Representative voltage responses to -100, -50, 0, 50 and 150 pA of step current injection in negative (red) and positive (green) engram cells. **d,** Average firing frequency corresponding to current injection (FI curve) (negative engrams: n=10, positive engrams: n=17) . Error bars correspond to standard error and no point of the curve was significantly different between the two populations. **e-g,** Suprathreshold characterization current-voltage responses of tagged hippocampal neurons. **e,** FI gain is the slope of the linear part of the FI curve shown in D, calculated as the change in firing frequency from the minimum firing to maximum firing divided by the injected current. **h-i,** Voltage and current step at which the neuron had at least one spike. **h,** Adaptation ratio is calculated as the mean inter spike interval (ISI) during the last 200 ms of spiking dividedthe mean ISI during the first 200 ms of spiking during a current step corresponding to 20 Hz firing rate. **i**, Input resistance for each neuron, calculated by averaging 25 trials for change in voltage divided by 25 pA injected while holding the cell at -70 mV. **j-l,** Analysis of spike shape of cells tagged by negative or positive experiences. **j,** Spike traces from all the negative and positive cells (Bold trace corresponds to average trace shape). **k,** Spike half width calculated as the time duration between two midpoints of the spikes. **l**, Deflection point of spike as identified by its first derivative. P-values were calculated based on independent t-test or Mann-Whitney test depending on normality of data. Mean $\pm$ SEM in bar graphs unless otherwise noted

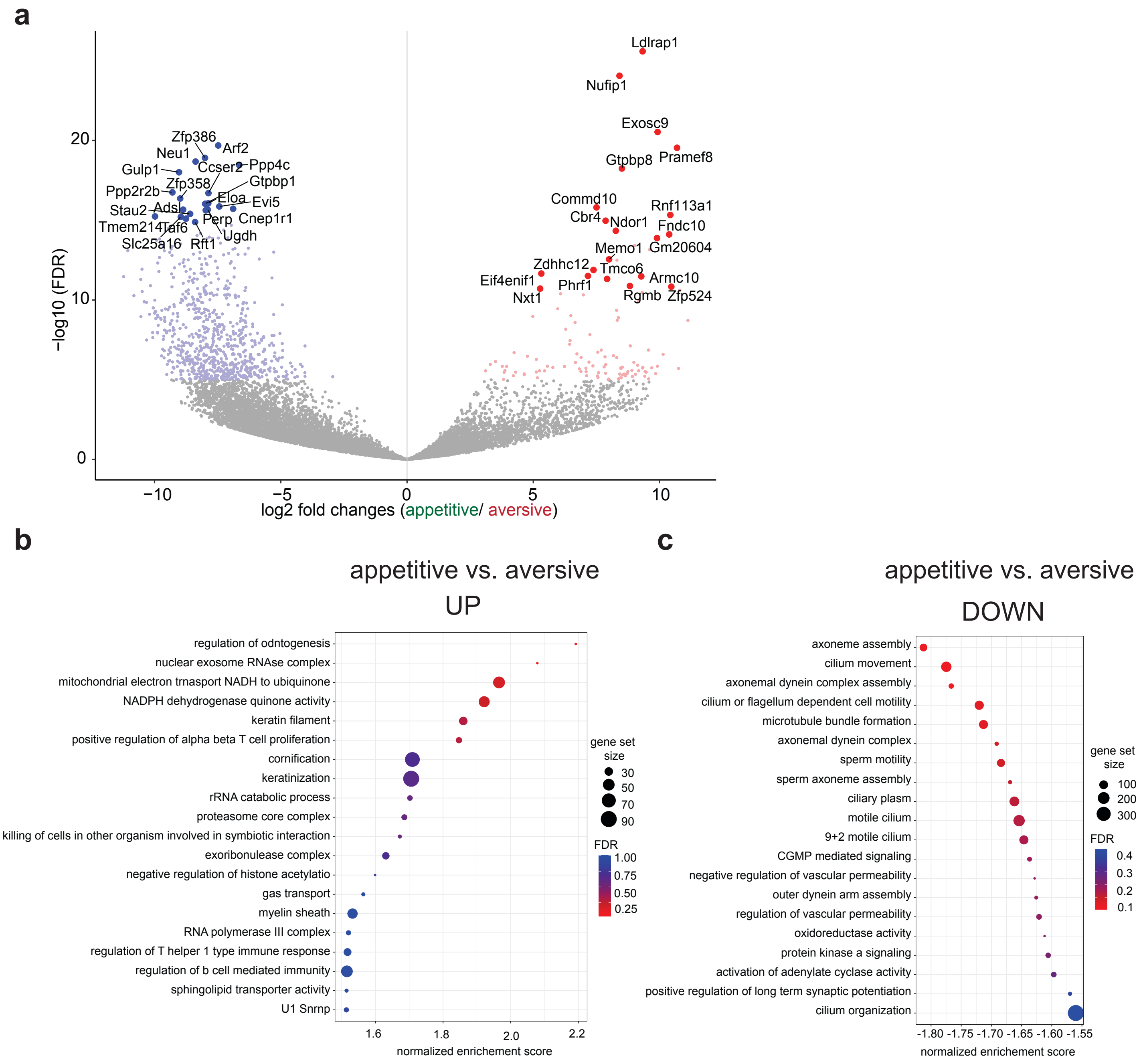
a
-log10 (FDR)
log2 fold changes (appetitive/ aversive)
Ldlrap1
Nufip1
Exosc9
Gtpbp8
Pramef8
Commd10
Rnf113a1
Cbr4
Ndor1
Fndc10
Memo1
Gm20604
Zdhhc12
Tmco6
Armc10
Eif4enif1
Phrf1
Nxt1
Rgmb
Zfp524
Zfp386
Arf2
Neu1
Ccser2
Ppp4c
Gulp1
Zfp358
Gtpbp1
Ppp2r2b
Eloa
Evi5
Stau2
Adsl
Perp
Cnep1r1
Tmem214
Taf6
Ugdh
Slc25a16
Rft1
b
appetitive vs. aversive
UP
regulation of odntogenesis
nuclear exosome RNAse complex
mitochondrial electron trnasport NADH to ubiquinone
NADPH dehydrogenase quinone activity
keratin filament
positive regulation of alpha beta T cell proliferation
cornification
keratinization
rRNA catabolic process
proteasome core complex
killing of cells in other organism involved in symbiotic interaction
exoribonulease complex
negative regulation of histone acetylatio
gas transport
myelin sheath
RNA polymerase III complex
regulation of T helper 1 type immune response
regulation of b cell mediated immunity
sphingolipid transporter activity
U1 Snrnp
normalized enrichement score
gene set size
FDR
c
appetitive vs. aversive
DOWN
axoneme assembly
cilium movement
axonemal dynein complex assembly
cilium or flagellum dependent cell motility
microtubule bundle formation
axonemal dynein complex
sperm motility
sperm axoneme assembly
ciliary plasm
motile cilium
9+2 motile cilium
CGMP mediated signaling
negative regulation of vascular permeability
outer dynein arm assembly
regulation of vascular permeability
oxidoreductase activity
protein kinase a signaling
activation of adenylate cyclase activity
positive regulation of long term synaptic potentiation
cilium organization
normalized enrichement score
gene set size
FDR

Supplementary Figure 5: RNA-seq analysis of hippocampus cells processing positive and negative memory engrams

a, Volcano plot with the relative fold change of gene expression in log 2 ratio and the FDR-adjusted p-value in log 2 ratio as X and Y axis showing the up and down regulated genes in positive vCA1 cells compared to negative vCA1 cells. Up or down regulated genes with FDR adjusted p-value less than 1e-5 plus at least four-fold differences were highlighted in either red or blue respectively. The gene names were displayed for the top 20 most significant protein coding genes as sorted by Wald statistics. **b,** Gene Set Enrichment Analysis of the up regulated pathways in positive engrams compared to negative engrams using Gene Ontology (GO) module. The size of the dot represents the gene count and the color of the dot indicates the FDR. Any pathway with a FDR smaller than 0.25 is considered as significantly enriched. **c,** GO analysis of down regulated pathways in positive engrams compared to negative engrams.

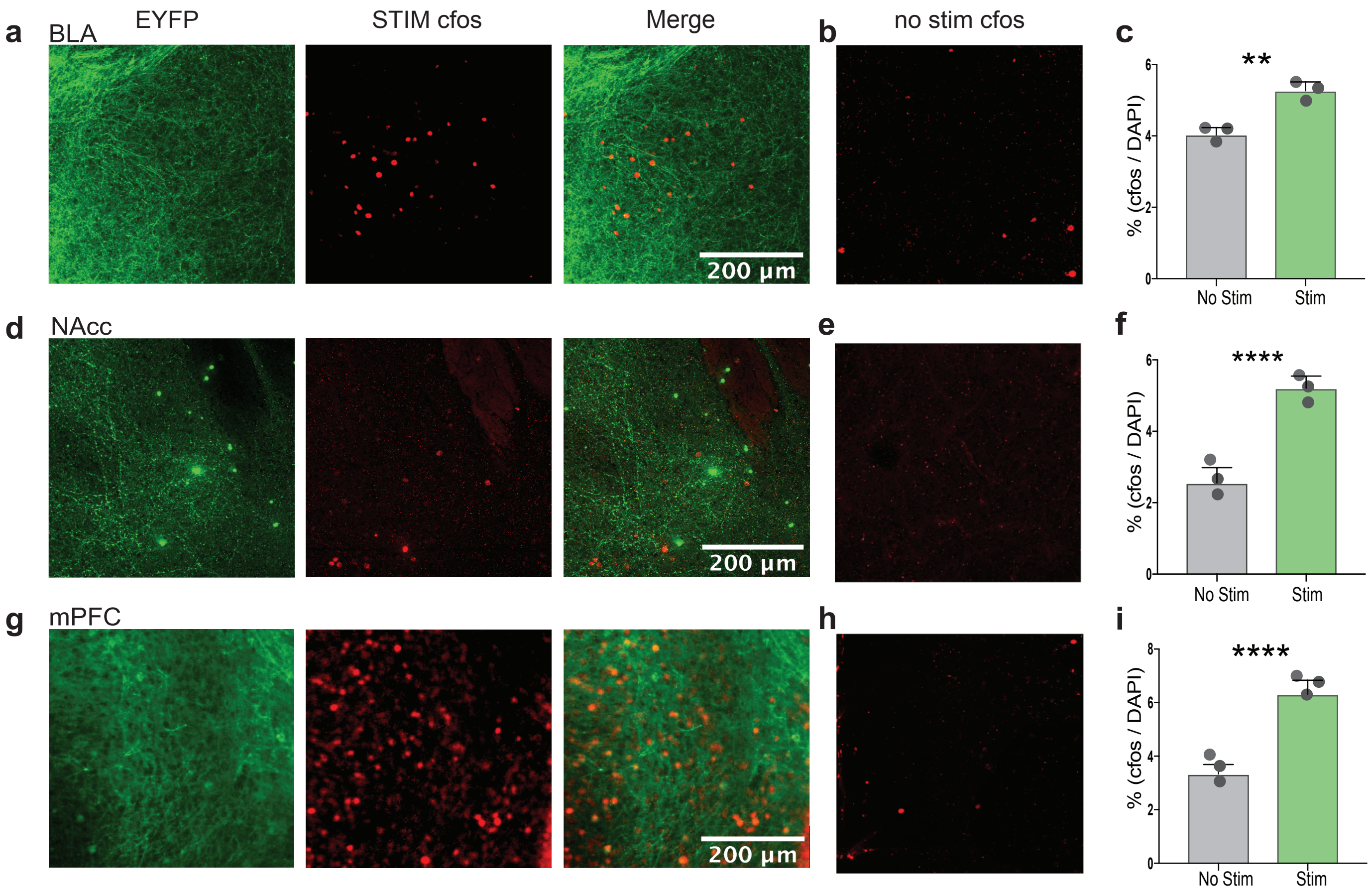

a
BLA
EYFP
STIM cfos
Merge
200 µm
b
no stim cfos
c
**
% (cfos / DAPI)
0
2
4
6
No Stim
Stim
d
NAcc
200 µm
e
f
****
% (cfos / DAPI)
0
2
4
6
No Stim
Stim
g
mPFC
200 µm
h
i
****
% (cfos / DAPI)
0
2
4
6
8
No Stim
Stim

Supplementary Figure 6: Terminal-specific optogenetic stimulation of vCA1 outputs leads to increased cFos in target areas

a, d, g, Representative images of ChR2-EYFP in BLA, NAc, and PFC terminals, respectively, and cFos expression after optical reactivation. **b, e, h**, Representative images of cFos without optogenetic reactivation of corresponding vCA1 terminals. **c, f, i**, Percent cFos/DAPI of BLA, NAc, and PFC non- stim vs stim groups (N=3, **P =0.0014, ****P < 0.0001 unpaired student's t-test, mean ± SEM in bar graphs unless otherwise noted)

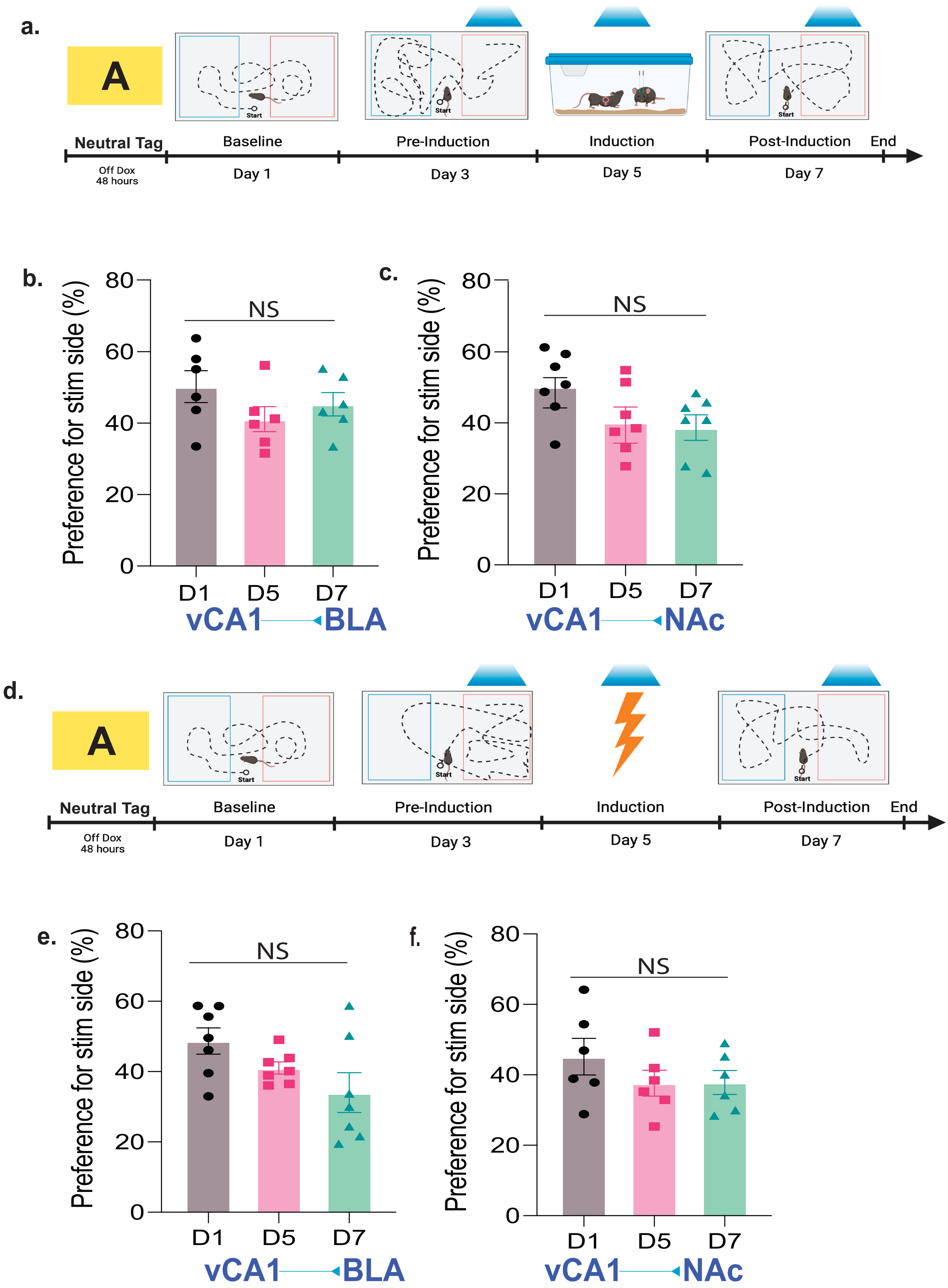
a.
A
Neutral Tag
Off Dox 48 hours
Baseline
Day 1
Pre-Induction
Day 3
Induction
Day 5
Post-Induction
Day 7
End
Start
b.
Preference for stim side (%)
0
20
40
60
80
NS
D1
D5
D7
vCA1
BLA
c.
Preference for stim side (%)
NS
D1
D5
D7
vCA1
NAc
d.
A
Neutral Tag
Off Dox 48 hours
Baseline
Day 1
Pre-Induction
Day 3
Induction
Day 5
Post-Induction
Day 7
End
e.
Preference for stim side (%)
NS
D1
D5
D7
vCA1
BLA
f.
Preference for stim side (%)
NS
D1
D5
D7
vCA1
NAc

Supplementary Figure 7: Activating cells processing a neutral memory does not lead to changes in avoidance or preference. a, Behavioral schedule in which vCA1 terminals are tagged in a neutral homecage experience (depicted as a yellow square during the off Dox period), followed by the induction protocol in which these outputs are activated concurrently during a positive experience in the BLA or NAc. Mice were injected with a virus cocktail consisting of cFos-tTa + TRE-ChR2-EYFP. **b-c,** Percent preference for the stimulation side when optically activating vCA1 terminals over the **b**, BLA or **c,** NAc at 20Hz during baseline (D1), pre-induction (D2), and post-induction (D3) tests. d, Behavioral schedule in which vCA1 terminals are tagged in a neutral homecage experience and during induction are reactivated during a negative (shock) experience. **e-f,** Percent preference for the stimulation side, neutral to negative, when optically activating vCA1 terminals over the **e,** BLA or **f,** NAc during D1, D5, and D7. (N=6-8; NS = not significant (P>0.05); repeated measures one-way ANOVA followed by Tukey's multiple comparison test, mean ± SEM in bar graphs unless otherwise noted).

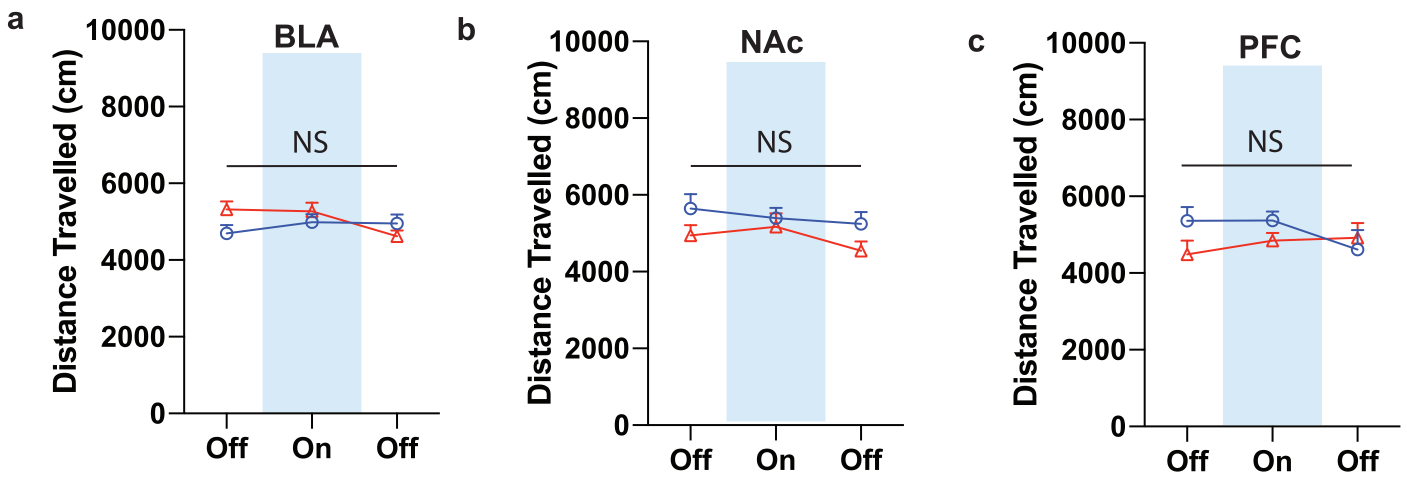
a
BLA
NS
Distance Travelled (cm)
0
2000
4000
6000
8000
10000
Off
On
Off
b
NAc
NS
Distance Travelled (cm)
0
2000
4000
6000
8000
10000
Off
On
Off
c
PFC
NS
Distance Travelled (cm)
0
2000
4000
6000
8000
10000
Off
On
Off

Supplementary Figure 8: Terminal-specific optogenetic stimulation of vCA1 outputs did not affect distance travelled in all groups. Stimulation of vCA1 terminals over the **a**, BLA (n=8), **b**, NAc (n=10), or **c**, PFC (n=9), had no effect on the distance traveled between light on or off epochs and across pre-induction or post-induction timepoints (NS, not significant; mean ± SEM in bar graphs unless otherwise noted)

Supplementary Table 1: Top 20 up-regulated and downregulated genes in negative vs. neutral cells

SYMBOL	GENE NAME
**Upregulated**	
Ccl4	chemokine (C-C motif) ligand 4
Ccl3	chemokine (C-C motif) ligand 3
Ccl12	chemokine (C-C motif) ligand 12
Ucp3	uncoupling protein 3 (mitochondrial, proton carrier)
Slitrk2	SLIT and NTRK-like family, member 2
Etnppl	ethanolamine phosphate phospholyase
Ccl7	chemokine (C-C motif) ligand 7
Adgrb2	adhesion G protein-coupled receptor B2
Rnf225	ring finger protein 225
Cd48	CD48 antigen
Fam198a	golgi associated kinase 1A
Cst7	cystatin F (leukocystatin)
Drp2	dystrophin related protein 2
Gli1	GLI-Kruppel family member GLI1
Tnf	tumor necrosis factor
Luzp2	leucine zipper protein 2
Kcnj9	potassium inwardly-rectifying channel, subfamily J, member 9
Cxcl2	chemokine (C-X-C motif) ligand 2
Kcnj16	potassium inwardly-rectifying channel, subfamily J, member 16
Myoc	myocilin
**Downregulated**	
Ldlrap1	low density lipoprotein receptor adaptor protein 1
Rbpms2	RNA binding protein with multiple splicing 2
Fbxo30	F-box protein 30
Dvl3	dishevelled segment polarity protein 3
Gtpbp8	GTP-binding protein 8 (putative)
Usb1	U6 snRNA biogenesis 1
Mrps27	mitochondrial ribosomal protein S27
Slc16a11	solute carrier family 16 (monocarboxylic acid transporters), member 11
Wdr31	WD repeat domain 31
Mark1	MAP/microtubule affinity regulating kinase 1
Lrrc41	leucine rich repeat containing 41
Osbpl5	oxysterol binding protein-like 5
Matn2	matrilin 2
Pramef8	Putative PRAME Family Member 24
Ccdc17	coiled-coil domain containing 17
Exosc9	exosome component 9
Gprc5c	G protein-coupled receptor, family C, group 5, member C
Ttll1	tubulin tyrosine ligase-like 1
Gbe1	glucan (1,4-alpha-), branching enzyme 1
Fance	Fanconi anemia, complementation group E

Supplementary Table 2: Top 20 upregulated and downregulated genes in positive vs. neutral cells

SYMBOL	GENE NAME
**Upregulated**	
Ccl4	chemokine (C-C motif) ligand 4
Ccl3	chemokine (C-C motif) ligand 3
Ccl12	chemokine (C-C motif) ligand 12
Etnppl	ethanolamine phosphate phospholyase
Papss2	3'-phosphoadenosine 5'-phosphosulfate synthase 2
Grik2	glutamate receptor, ionotropic, kainate 2 (beta 2)
Csmd2	CUB and Sushi multiple domains 2
Igdcc4	immunoglobulin superfamily, DCC subclass, member 4
F3	coagulation factor III
Kcnc4	potassium voltage gated channel, Shaw-related subfamily, member 4
Gria2	glutamate receptor, ionotropic, AMPA2 (alpha 2)
Rbp4	retinol binding protein 4, plasma
Slc1a2	solute carrier family 1 (glial high affinity glutamate transporter), member 2
Tnf	tumor necrosis factor
Phactr3	phosphatase and actin regulator 3
Gli1	GLI-Kruppel family member GLI1
Scg3	secretogranin III
Hrh1	histamine receptor H1
Ftmt	ferritin mitochondrial
Pdzph1	PDZ and pleckstrin homology domains 1
**Downregulated**	
Perp	PERP, TP53 apoptosis effector
Hemk1	HemK methyltransferase family member 1
Ppp4c	protein phosphatase 4, catalytic subunit
Hprt	hypoxanthine guanine phosphoribosyl transferase
Pcolce	procollagen C-endopeptidase enhancer protein
Sdf2l1	stromal cell-derived factor 2-like 1
Arf2	ADP-ribosylation factor 2
Trpv4	transient receptor potential cation channel, subfamily V, member 4
Evi5	ecotropic viral integration site 5
Fbxw11	F-box and WD-40 domain protein 11
Spint2	serine protease inhibitor, Kunitz type 2
Slc31a1	solute carrier family 31, member 1
Rfng	RFNG O-fucosylpeptide 3-beta-N-acetylglucosaminyltransferase
Tmem214	transmembrane protein 214
Eloa	elongin A
Ttc9	tetratricopeptide repeat domain 9
Ugdh	UDP-glucose dehydrogenase
Acd	adrenocortical dysplasia
Adsl	adenylosuccinate lyase
Zdhhc1	zinc finger, DHHC domain containing 1

Supplementary Table 3: Top 20 upregulated and downregulated genes in positive vs. negative cells

SYMBOL	GENE NAME
**Upregulated**	
Ldlrap1	low density lipoprotein receptor adaptor protein 1
Nufip1	nuclear fragile X mental retardation protein interacting protein 1
Exosc9	exosome component 9
Pramef8	Putative PRAME Family Member 24
Gtpbp8	GTP-binding protein 8 (putative)
Commd10	COMM domain containing 10
Rnf113a1	ring finger protein 113A1
Cbr4	carbonyl reductase 4
Ndor1	NADPH dependent diflavin oxidoreductase 1
Fndc10	fibronectin type III domain containing 10
Gm20604	predicted gene 20604
Memo1	mediator of cell motility 1
Zdhhc12	zinc finger, DHHC domain containing 12
Eif4enif1	eukaryotic translation initiation factor 4E nuclear import factor 1
Phrf1	PHD and ring finger domains 1
Armc10	armadillo repeat containing 10
Tmco6	transmembrane and coiled-coil domains 6
Rgmb	repulsive guidance molecule family member B
Zfp524	zinc finger protein 524
Nxt1	NTF2-related export protein 1
**Downregulated**	
Arf2	ADP-ribosylation factor 2
Zfp386	zinc finger protein 386 (Kruppel-like)
Neu1	neuraminidase 1
Ppp4c	protein phosphatase 4, catalytic subunit
Gulp1	GULP, engulfment adaptor PTB domain containing 1
Ppp2r2b	protein phosphatase 2, regulatory subunit B, beta
Ccser2	coiled-coil serine rich 2
Zfp358	zinc finger protein 358
Eloa	elongin A
Gtpbp1	GTP binding protein 1
Evi5	ecotropic viral integration site 5
Cnep1r1	CTD nuclear envelope phosphatase 1 regulatory subunit 1
Ugdh	UDP-glucose dehydrogenase
Adsl	adenylosuccinate lyase
Perp	PERP, TP53 apoptosis effector
Stau2	staufen double-stranded RNA binding protein 2
Tmem214	transmembrane protein 214
Slc25a16	solute carrier family 25 (mitochondrial carrier, Graves disease autoantigen), member 16
Taf6	TATA-box binding protein associated factor 6
Rft1	RFT1 homolog

Supplementary Table 4: GeneMANIA Network_shared protein domains

Shared protein domains	Links
A-amylase/branching_C	Alpha-amylase/branching enzyme, C-terminal all beta
Aminotrans_3	Aminotransferase class-III
Chemokine_CC_CS	CC chemokine, conserved site
Chemokine_IL8-like_dom	Chemokine interleukin-8-like domain
Cyclic_Pdiesterase	Cyclic phosphodiesterase
Dishevelled_fam	Dishevelled family
Dishevelled_protein_dom	Dishevelled protein domain
DIX	DIX domain
ExoRNase_PH_dom2	Exoribonuclease, phosphorolytic domain 2
F-box_dom	F-box domain
Fringe	Fringe
Fumarate_lyase_fam	Fumarate lyase family
Glu/Gly-bd	Ionotropic glutamate receptor, L-glutamate and glycine-binding domain
GPCR_2_brain_angio_inhib	GPCR, family 2, brain-specific angiogenesis inhibitor
Ig_E-set	Immunoglobulin E-set
Interleukin_8-like_sf	Chemokine interleukin-8-like superfamily
Iono_rcpt_met	Ionotropic glutamate receptor, metazoa
Iontro_rcpt	Ionotropic glutamate receptor
IRK_C	Inward rectifier potassium channel, C-terminal
K_chnl_inward-rec_Kir	Potassium channel, inwardly rectifying, Kir
K_chnl_volt-dep_Kv3	Potassium channel, voltage dependent, Kv3
KA1_dom	Kinase associated domain 1 (KA1)
Kir_TM	Potassium channel, inwardly rectifying, transmembrane domain
L-Aspartase-like	L-Aspartase-like
Matrilin_cc_sf	Matrilin, coiled-coil domain superfamily
Matrilin_coiled-coil_trimer	Matrilin, coiled-coil trimerisation domain
Mit_uncoupling_UCP-like	Mitochondrial carrier UCP-like
Na-dicarboxylate_symporter	Sodium:dicarboxylate symporter
Na:dicarbo_symporter_sf	Sodium:dicarboxylate symporter superfamily
PRibTrfase_dom	Phosphoribosyltransferase domain
RPEL_repeat	RPEL repeat
Small_mtfrase_dom	Methyltransferase small domain
TNF	Tumour necrosis factor
TRPV1-4_channel	Transient receptor potential cation channel subfamily V member 1-4

Supplementary Table 5: Genome position annotation of DMCs

Genome position of DMCs in Negative vs Neutral

Genome position	Number of DMCs	Distribution
3' UTR	27	1.39%
5' UTR	1	0.05%
Exon	29	1.50%
Intergenic	1008	51.99%
Intron	779	40.18%
Non-coding	30	1.55%
Promoter	33	1.70%
TTS	32	1.65%

Genome position of DMCs in Positive vs Neutral

Genome position	Number of DMCs	Distribution
3' UTR	29	0.93%
5' UTR	0	0.00%
Exon	89	2.86%
Intergenic	1514	48.57%
Intron	1305	41.87%
Non-coding	68	2.18%
Promoter	60	1.92%
TTS	52	1.67%

Promoter defined from -1kb to transcription start site(TSS).

TSS: Transcription termination site, defined from -100bp to +1kb
