# Supplementary material for: Hippocampal cells segregate positive and negative engrams
Source: Commun Biol. 2022 Sep 26;5:1009. doi: 10.1038/s42003-022-03906-8 (PMC9512908; doi:10.1038/s42003-022-03906-8)
Supplement: Supplementary file 2 — Description of Additional Supplementary Files [file 42003_2022_3906_MOESM2_ESM.pdf]

File Name: Supplementary Data 1
Description: Differently-methylated genes

File Name: Supplementary Data 2
Description: Source data underlying main figures.
