# Supplementary material for: Hippocampal cells segregate positive and negative engrams
Source: Commun Biol. 2022 Sep 26;5:1009. doi: 10.1038/s42003-022-03906-8 (PMC9512908; doi:10.1038/s42003-022-03906-8)
Supplement: Supplementary file 5 — Reporting Summary [file 42003_2022_3906_MOESM5_ESM.pdf]

nature portfolio

Corresponding author(s): Steve Ramirez

Last updated by author(s): Aug 25, 2022

Reporting Summary

Nature Portfolio wishes to improve the reproducibility of the work that we publish. This form provides structure for consistency and transparency in reporting. For further information on Nature Portfolio policies, see our Editorial Policies and the Editorial Policy Checklist.

Statistics

For all statistical analyses, confirm that the following items are present in the figure legend, table legend, main text, or Methods section.

n/a	Confirmed	
☐	☒	The exact sample size (*n*) for each experimental group/condition, given as a discrete number and unit of measurement
☐	☒	A statement on whether measurements were taken from distinct samples or whether the same sample was measured repeatedly
☐	☒	The statistical test(s) used AND whether they are one- or two-sided<br>*Only common tests should be described solely by name; describe more complex techniques in the Methods section.*
☒	☐	A description of all covariates tested
☐	☒	A description of any assumptions or corrections, such as tests of normality and adjustment for multiple comparisons
☐	☒	A full description of the statistical parameters including central tendency (e.g. means) or other basic estimates (e.g. regression coefficient) AND variation (e.g. standard deviation) or associated estimates of uncertainty (e.g. confidence intervals)
☐	☒	For null hypothesis testing, the test statistic (e.g. *F*, *t*, *r*) with confidence intervals, effect sizes, degrees of freedom and *P* value noted<br>*Give P values as exact values whenever suitable.*
☒	☐	For Bayesian analysis, information on the choice of priors and Markov chain Monte Carlo settings
☒	☐	For hierarchical and complex designs, identification of the appropriate level for tests and full reporting of outcomes
☒	☐	Estimates of effect sizes (e.g. Cohen's *d*, Pearson's *r*), indicating how they were calculated

Our web collection on statistics for biologists contains articles on many of the points above.

Software and code

Policy information about availability of computer code

Data collection	For real time place preference EthoVision software via Noldus USB-IO Box triggers a stimulus generatior (STG-4008, multichannel systems). 6A video camera (Activeon CX LCD Action Camera) recorded each session and presented raw data of time spent on stimulated side.<br>All fluorescent images were obtained at 20X using a Zeiss confocal microscope (LSM-800) using Zen Blue 2.3 software. Cell counts were done manually using Image J/Fiji software (version 2.1.0; https://imagej.nih.gov/ij/).
Data analysis	Data was analyzed using parametric statistics (Prism version 9.2.0) following tests of normality and homogeneity of variances. Standard T-Tests or One-Way ANOVAs were run. If ANOVA was run, post hoc analysis was assessed using Tukey's Mulitple Comparisons test.

For manuscripts utilizing custom algorithms or software that are central to the research but not yet described in published literature, software must be made available to editors and reviewers. We strongly encourage code deposition in a community repository (e.g. GitHub). See the Nature Portfolio guidelines for submitting code & software for further information.

Data

Policy information about availability of data

All manuscripts must include a data availability statement. This statement should provide the following information, where applicable:

- Accession codes, unique identifiers, or web links for publicly available datasets
- A description of any restrictions on data availability
- For clinical datasets or third party data, please ensure that the statement adheres to our policy

The data supporting the findings from this study are available from the corresponding author upon request.

Field-specific reporting

Please select the one below that is the best fit for your research. If you are not sure, read the appropriate sections before making your selection.

☒ Life sciences ☐ Behavioural & social sciences ☐ Ecological, evolutionary & environmental sciences

For a reference copy of the document with all sections, see nature.com/documents/nr-reporting-summary-flat.pdf

Life sciences study design

All studies must disclose on these points even when the disclosure is negative.

Sample size	No statistical test was used to predetermine group sizes. Group sizes were selected based on what is standard for the field.
Data exclusions	Mice were excluded if viral injections were mistargeted or unilateral or if they were euthanized for health reasons (e.g., head caps came off, mice lethargic / not grooming, etc)
Replication	Behavioral data was replicated in two different institutes and two different vivariums. The behavioral data was also replicated by three different individuals. For RNA-Seq, data was replicated three times and batch-effects were controlled for. For cell counting right and left hemisphere counts were combined.
Randomization	All animals were randomly assigned to experimental groups upon arrival. During behavioral the region of preference or aversion was counterbalanced.
Blinding	All tests were run blind to condition / group assignments. Cell counts and behavioral scoring were also run blind.

Reporting for specific materials, systems and methods

We require information from authors about some types of materials, experimental systems and methods used in many studies. Here, indicate whether each material, system or method listed is relevant to your study. If you are not sure if a list item applies to your research, read the appropriate section before selecting a response.

Materials & experimental systems

n/a	Involved in the study	
☐	☒	Antibodies
☒	☐	Eukaryotic cell lines
☒	☐	Palaeontology and archaeology
☐	☒	Animals and other organisms
☒	☐	Human research participants
☒	☐	Clinical data
☒	☐	Dual use research of concern

Methods

n/a	Involved in the study	
☒	☐	ChIP-seq
☐	☒	Flow cytometry
☒	☐	MRI-based neuroimaging

Antibodies

Antibodies used	1:1000 guinea anti-c-Fos (SySy 226-004), 1:1000 chicken anti-GFP (Invitrogen a10262), 1:1000 rabbit anti-RFP (Rockland, 200-302-379). Secondary antibodies 1:200 Alexa 555 goat-anti-Rabbit (Invitrogen; A-21429), 1:200 647 goat-anti-Guinea (Invitrogen; A-21450), 1:200 488 goat-anti-Chicken (Invitrogen; A-11039)
Validation	All antibodies are widely used and have been used in previous publications.

Animals and other organisms

Policy information about studies involving animals; ARRIVE guidelines recommended for reporting animal research

Laboratory animals	Wild-type male c57BL/6 mice (~39 days of age; Charles River Labs) 20–22 g upon arrival.<br>Wild-type female c57BL/6 mice (~39 days of age; Charles River Labs) 18–20 g upon arrival.<br>Two Fos[2A-iCreER](TRAP2) breeding pairs (The Jackson Laboratory; 030323) to maintain an in-house colony.<br>Regular light cycle 12:12 h light–dark; temperature and humidity-controlled colony room. Fed 40 mg/kg DOX diet (Bio-Serv, product F4159, Lot 226766) - ad libitum access to food and water.
Wild animals	N/A
Field-collected samples	N/A

Ethics oversight	Experimental procedures were conducted in accordance with protocol 2018000579 approved by the Institutional Animal Care and Use Committee at Boston University.

Note that full information on the approval of the study protocol must also be provided in the manuscript.

Flow Cytometry

Plots

Confirm that:

☒ The axis labels state the marker and fluorochrome used (e.g. CD4-FITC).

☒ The axis scales are clearly visible. Include numbers along axes only for bottom left plot of group (a 'group' is an analysis of identical markers).

☒ All plots are contour plots with outliers or pseudocolor plots.

☒ A numerical value for number of cells or percentage (with statistics) is provided.

Methodology

Sample preparation	Five-week old male mice labeled with ChR2-YFP transgene1 after conditioning were euthanized by isoflurane. Mouse brains were rapidly extracted, and the hippocampal regions were isolated by microdissection. Eight mice were pooled by each experimental condition. Single cell suspension was prepared according to the guideline of Adult Brain Dissociation Kit (Miltenyi Botec, Cat No: 13-107-677). Briefly, the hippocampal samples were incubated with digestion enzymes in the C Tube placed on the gentleMACS Octo Dissociator with Heaters with gentleMACS Program: 37C_ABDK_01. After termination of the program, the samples were applied through a MACS SmartStrainer (70 µm). Then a debris removal step and a red blood cell removal step were applied to obtain single cell suspension
Instrument	BD FACSAria cell sorter.
Software	The analysis software with BD FACSAria cell sorter.
Cell population abundance	The EYFP+ cell population is ~0.5-0.6% of total single cell suspension.
Gating strategy	The single cell suspension prepared from wild type mice was used a negative control to identify the EFYP+ cells from experimental samples. The example gating strategy is shown on Figure 3B.

☒ Tick this box to confirm that a figure exemplifying the gating strategy is provided in the Supplementary Information.
